# Analyzing the Influence of Anthropogenic Heat on Groundwater Using Remote-Sensing and In Situ Data

**DOI:** 10.3390/s25206351

**Published:** 2025-10-14

**Authors:** Surya Deb Chakraborty, M. Sami Zitouni, Saeed Al Mansoori, P. Jagadeeswara Rao, K. Mruthyunjaya Reddy

**Affiliations:** 1 College of Engineering and Information Technology, University of Dubai, Dubai 14143, United Arab Emirates; mzitouni@ud.ac.ae; 2 Mohammed Bin Rashid Space Center (MBRSC), Dubai 211833, United Arab Emirates; saeed.almansoori@mbrsc.ae; 3 Department of Geo-Engineering & Resource Development Technology, Andhra University, Visakhapatnam 530003, India; pjr_geoin@rediffmail.com; 4 National Remote Sensing Centre, ISRO, DOS, Government of India, Hyderabad 500037, India; kmruthyu@yahoo.com

**Keywords:** land surface temperature (LST), remote sensing, urban heat island (UHI), Landsat, thermal band, land use/land cover (LULC)

## Abstract

**Highlights:**

**What are the main findings?**

- Bangalore’s built-up area increased from 7.61% to 28.78% during the period from 1999 to 2017, causing a rise in the land surface temperature (LST) and anthropogenic heat flux (>65 W/m^2^). There was a rise in temperature (LST) of ~6 °C in green areas and >3 °C in urban areas.
- Groundwater temperature was strongly correlated with heat flux (R^2^ = 0.83) and with LST (R^2^ = 0.78), indicating subsurface warming due to urbanization.

**What is the implication of the main finding?**

- Sustainable urban planning is needed to reduce the heat stress by increasing the extent of green spaces, adopting cool roofing, and improving water resource management.
- Incorporating urban heat assessment in policies can enhance thermal comfort, reduce heat-related illness and mortality, mitigate groundwater warming, and ensure long-term water and energy sustainability in rapidly urbanizing cities, such as Bangalore.

**Abstract:**

The continuous expansion of impervious surfaces replacing the vegetation cover and surface water areas increases urban heating. Such heating leads to downward heat transfer and latent heat flux from the surface to subsurface aquifers. This study used Landsat optical and thermal satellite data for land use/land cover (LULC), land surface temperature (LST), and anthropogenic heat flux (H_as_) change mapping in Bangalore City, India. The in situ sensor-based land surface temperature (LST) and groundwater temperature (GWT) measurements were used to validate the study outcome. A minor difference was observed between the satellite data and the in situ LST due to the differential data acquisition time. The built-up area increased from 7.61% to 28.78% from 1999 to 2017 at the cost of the green cover and the extent of waterbodies. Therefore, LST change was higher in green cover areas (~6 °C LST) than in urban areas (>3 °C). The anthropogenic heat fluxes increased significantly (above 65 W/m^2^) during the study period. The in situ GWT was strongly correlated with the H_as_ (R^2^ = 0.83) and LST (R^2^ = 0.78). The study highlights the nature of urban expansion in Bangalore City, India, and its impact on LST, H_as_, and GWT. The observed changes in land use practices with urban heat indicators at 30 m scale can be used for sustainable land use planning to improve the thermal comfort of the city, preserving the urban ecosystems. The high collinearity between satellite-data-derived LST, H_as_, and GWT can be used for periodic monitoring at seasonal and annual scales using the Landsat data, which can be important inputs for land use planners and policymakers.

## 1. Introduction

The rapid horizontal and vertical expansion of urban areas, widespread use of cooling systems, reduction in green and water spaces, and the growth of impervious surfaces are significantly altering microclimatic conditions in cities. The rise in air temperature and land surface temperature (LST) in urban areas, compared with the surrounding rural zones, creates distinct local climate zones that are shaped by urban morphology [1]. This temperature differential forms the urban heat island (UHI) effect, reflecting contrasts in biophysical and climatic characteristics between urban and rural areas. The low albedo and high thermal capacity of asphalt concrete, which is commonly used in urban pavements, can raise LSTs to over 50 °C during peak summer days [2]. Additionally, the conversion of forests, croplands, and peri-urban green space into built-up areas, combined with industrial growth, loss of waterbodies, and increased vehicular emissions, has reduced evapotranspiration and increased surface runoff [3,4]. Surface emissivity, which represents the efficiency with which the earth’s surface emits thermal radiation, plays a key role in balancing incoming solar and outgoing thermal energy. The UHI effect is primarily driven by increased sensible heat flux from the urban surface to the atmosphere. This includes both solar heat radiation absorbed and re-emitted by land surfaces and anthropogenic heat released through human activities, such as industrial processes, transportation, and building energy use. Together, these components contribute to elevated urban temperatures. Anthropogenic heat flux is often used as a quantitative indicator of the intensity of the UHI effect [5].

LST and surface emissivity are critical parameters for energy budget analysis, urban climate studies, and evaluating the impacts of LULC change. The main contributors to urban heating include urban geometry (clusters of tall buildings trap heat and restrict airflow), anthropogenic emissions from vehicles and industry, and the widespread use of heat-retaining materials. These indicators also support the development of nature-based solutions (NBS) and contribute to achieving Sustainable Development Goals (SDGs) [6,7]. According to IPCC AR6 [8], urban geometry makes the largest contribution to the UHI effect, with an average increase of 1.25 °C. This is followed by anthropogenic emissions, which contribute about 1.0 °C, and heat-absorbing urban materials, which add approximately 0.7 °C. In contrast, preserving waterbodies and green cover, such as parks and gardens, helps mitigate the UHI effect. These features can potentially reduce average urban temperatures by up to 0.8 °C and 1.6 °C, respectively. The UHI effect has serious implications for human health and well-being. It increases the risk of heat-related illnesses and mortality among millions of people living in urban environments globally [9].

Thus, monitoring land use and land cover (LULC) changes, LST, and other surface characteristics is essential for urban planners and architects to promote sustainable development and improve urban livability. Previous studies recommended monitoring land use practices in urban settings for urban landscape development planning; for example, Balasubramanian et al. [10] studied land use practices in Kochi City, Kerala, to identify the potential for tree-based restoration aiming to mitigate urban heating. Gómez et al. [11] emphasized the role of green cover in enhancing thermal comfort and retaining pollutants in Valencia, Spain, and estimated the green cover required to maintain urban thermal comfort.

Accurate measurement of LST across various parts of an urban area is essential to understand the influence of anthropogenic activities on microclimatic conditions. Several instruments and sensors are used for LST measurement, including infrared temperature sensors, pyrgeometers (for long-wave radiation measurements), and infrared cameras equipped with thermocouples [12]. However, long-term and spatially extensive in situ data required for monitoring the impact of LULC change on LST are often limited or unavailable, particularly in developing countries. In contrast, satellite-based imaging sensors provide valuable indicators for such studies, including vegetation cover, built-up area, waterbodies, surface albedo, land surface emissivity, and LST. Satellite missions such as Landsat provide optical and thermal imagery that are critical for evaluating surface temperature, heat flux, and their linkages with land surface changes over the past five decades. Landsat thermal images, captured at 16-day intervals, are particularly useful for understanding energy interactions at monthly and seasonal scales at high spatial resolution (less than 100 m) across the globe. In comparison, thermal data from VIIRS, MODIS, and Sentinel-3 have a coarser resolution of 750 m, 1 km, and 1 km, respectively, at a temporal interval of 1–4 days. Although VIIRS and MODIS data have been available for the past two decades, Sentinel-3 provides recent data. Due to their coarser resolution, these are more useful for studies at the regional, national, and global levels.

Martin et al. [13] compared in situ LST measurements with multiple satellite-derived LST values under various surface conditions, such as anisotropy, topography, and land cover. Their findings showed greater accuracy during daytime observations, with deviations of ±2 K, compared with night-time measurements. Almeida et al. [14] reviewed global satellite-based UHI assessments and reported that Landsat and MODIS are the two most used LST products in relation to LULC monitoring. James et al. [15] discussed the use of thermal remote sensing in urban climate studies, emphasizing that while it is widely applied for UHI analysis, progress has been slow in moving beyond simple thermal pattern descriptions and basic correlations. Although numerous studies analyzed LULC change with LST change in various continents, studies on anthropogenic heat flux are limited, which significantly contributes to both elevated atmospheric and subsurface groundwater temperatures. In addition to LST, subsurface urban infrastructure, including heating systems in temperate and sub-temperate zones, has a strong influence on groundwater temperatures in shallow aquifers [16,17]. Benz et al. [18] investigated the relationship between the UHI effect and subsurface urban heat islands (SUHIs) in German cities using satellite-derived LST and groundwater temperature data. They proposed a novel method for estimating urban groundwater temperature (GWT) based on LST, building density, and elevated basement temperatures. Global research further indicates that shallow groundwater temperatures can be estimated using surface air temperature, with adjustments made based on insulation and latent heat flow [19]. Hemmerle et al. [20] used satellite-derived LST in Paris to examine thermal interactions between the surface and shallow aquifers. Their results highlighted the role of seasonal snow cover, insulation, and heating systems in regulating downward heat transfer and latent heat flux in urban environments.

India houses more than 1.4 billion people, with several mega-cities and rapidly expanding urban areas. Several studies have demonstrated the potential use of satellite data in monitoring urban expansion and the associated LST increases in Indian cities [21,22,23]. However, limited studies have been conducted to assess the impact of urban growth and LST on the GWT in India through satellite remote sensing. Moreover, there are limited resources available for measuring GWT in India. Chakraborty et al. [24], for instance, examined the impact of LULC changes and anthropogenic disturbances on LST in Delhi. Their study revealed that urbanization and industrialization led to an increase in LST of 1.4 °C and in anthropogenic heat flux of 38 W/m^2^. However, such assessments are not available for other important Indian cities.

Bangalore City, the capital of Karnataka, India, faces numerous environmental challenges resulting from rapid economic development. These include significant LULC changes, deforestation, urbanization, water pollution, and land encroachment [25]. The expansion of information technology parks and special economic zones has triggered increased population migration and rapid urban sprawl. The growing extent of impermeable surfaces, such as roads, buildings, and sewer systems, has altered the city’s physical geography, exacerbating the UHI effect. This study focuses on assessing the impact of LULC changes on LST and on how both LST and anthropogenic heat flux influence groundwater temperatures in Bangalore. It integrates satellite remote sensing with field measurements to evaluate the contribution of anthropogenic and natural heat sources, primarily through sensible heat flux. The study also aims to evaluate the reliability of Landsat data in analyzing surface emissivity and LST by validating them against in situ measurements.

## 2. Materials and Methods

### 2.1. Study Area

**Figure 1:**
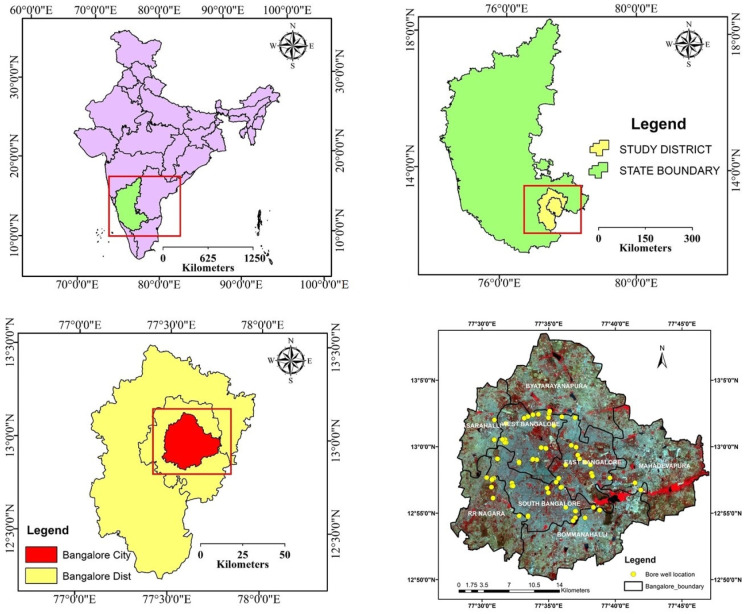
Location map of the Karnataka district in India, the Bangalore district in Karnataka, Bangalore City, and in situ measurement (groundwater temperature) sites on the Landsat satellite image.

The study was conducted in Bangalore City (modern-day Bengaluru) and its surrounding areas, located between latitudes 12°39′ N and 13°18′ N and longitudes 77°22′ E and 77°52′ E. The city sits at an elevation of approximately 894 m and occupies an area of around 2072 km^2^ (Figure 1). In 2006, Bangalore’s administrative boundaries were expanded to include eight neighboring municipal bodies. With a population of over 7 million, Bangalore is the sixth largest metropolitan area in India. The region experiences a tropical climate, characterized by wetter summers and drier winters. Summer temperatures typically range from 18 °C to 38 °C, while winter temperatures range from 12 °C to 25 °C. The area receives rainfall predominantly from the southwest monsoon and a smaller contribution from the northeast monsoon, with the average annual rainfall between 800 mm and 900 mm. The city’s vegetation includes deciduous trees, tropical evergreen species, fruit trees, agricultural crops, and grassland vegetation. Bangalore is also known for its historic gardens, such as Lalbagh and Cubbon Park, as well as numerous lakes and tanks that support a variety of wildlife [26,27]. However, the region’s physical and cultural landscapes have undergone substantial transformation due to industrialization and rapid urban expansion [27]. According to the major aquifer data available in the India-WRIS portal (https://indiawris.gov.in/wris/#/geoSpatialData, accessed on 8 September 2025), there is one major aquifer (namely the Basement Gneissic Complex), indicating the uniformity of the aquifer system in the study area.

### 2.2. Input Data

Satellite data: Seasonal heat fluxes were estimated using Landsat-TM 5 and Landsat-8 datasets for different LULC classes in the study area for the years 1999, 2009, and 2017/2018. The satellite imagery was pre-processed using Fast Line-of-Sight Atmospheric Analysis of Spectral Hypercubes (FLAASH) for atmospheric correction. This was followed by spatial sub-setting to match the geographic extent of the study area.

In situ data: Groundwater temperature (GWT) data from 77 borewells were collected in 2009 through a field survey to examine the relationship between GWT and anthropogenic heat flux. The GWT measurements were obtained using a data logger at a depth of ~100 feet below the ground surface on 14 April 2009. In addition, the LST data were collected using an infrared thermometer on 24 and 25 April 2017. These measurements were taken near borewell locations (where data were collected in 2009) across various LULC categories to ensure consistency between surface and subsurface thermal observations. LST values were sampled at 80 sites in 2017, between 10:00 a.m. and 1:00 p.m., to capture peak daytime heating conditions. The India Meteorological Department (IMD) climate data and SRTM-DEM were used to estimate the various parameters of anthropogenic heat flux.

Table 1 summarizes the datasets used in the present study.

### 2.3. Methodology

#### 2.3.1. LULC Classification

Land use/land cover (LULC) classification was carried out to generate LULC maps for the years 1999, 2009, and 2017/2018. Four major LULC classes were identified and mapped: built-up areas, waterbodies, vegetation, and agriculture/plantations. The vegetation class represents the areas dominated by tree cover, including parks, forest patches, and green corridors. Training data points for classification were generated through visual interpretation of satellite imagery. For each LULC class, more than 100 training samples were identified to ensure accurate classification. A supervised classification approach using the maximum likelihood classifier (MLC) was applied to develop LULC maps for all three time periods.

#### 2.3.2. Land Surface Temperature and Anthropogenic Heat Flux (H_as_) Estimation

Heat fluxes were estimated for the winter and summer seasons. Thermal bands of Landsat-TM 5 (band 6) and Landsat-8 (band 10) imagery were used, which recorded the effective at-sensor temperatures of the viewed earth-atmosphere system under an assumption of unity emissivity and using pre-launch calibration constants [28]. The major equations are added here in the Methodology section, wherein detailed methodology for estimating anthropogenic heat flux published in our previous study can be referred to (Chakraborty et al. [24]).

The digital number (DN) of the thermal infrared band is converted into spectral radiance (Lλ) using the calibration constants, which were used to estimate LST (T) using the surface emissivity values.

(1) $$T=\frac{k_{2}}{In\left(\frac{\varepsilon k_{1}}{L_{\lambda }}\right)+1}\;\text{where}\;L\lambda =c\times \mathrm{DN}+d$$

where for Landsat-TM 5: K1 = 607.76, K2 = 1260.56, for Landsat-TM 8: K1 = 774.88, K2 = 1260.56, L_λ_ = mWcm^−2^ sr^−1^ µm^−1^; c = band’s gain factor, d = band’s scale factor [band-specific values are obtained from image metadata file], and ε is the land surface emissivity.

The energy balance equation was used to compute the heat flux using the following equation:

(2) $$R_{n}=LE+G+H$$

where Rn is net radiation; LE is the latent heat flux; G is the ground heat flux; and H is the sensible heat flux. The evapotranspiration of the land surface creates latent heat, while sensible heat is transferred from the land surface to the atmosphere. However, over urban areas, in addition to the net radiation, the anthropogenic heat discharge (A), mainly from urban structures and transportation, adds to the heat flux component, described as

(3) $$R_{n}+A=LE+G+H$$

Net radiation is the sum of the incoming and outgoing short- and long-wave components. Net radiation R_n_ is the dominant term in the energy balance equation, since it represents the energy source that must be balanced by the thermodynamic equilibrium of the other terms.

Ground heat flux (G) can be presented by the amount of heat transmitted per unit area per unit time, computed as

(4) $$G=\left(0.1-0.042h\right)R_{n}$$

where h is the reference height.

The sensible heat flux is computed based on the theory of mass transport of heat and momentum between the surface and the near-surface environment, which is expressed as

(5) $$H=\rho C_{P}\frac{T_{s}-T_{a}}{r_{a}}$$

where ρ is the air density; Cp is specific heat; Ts is surface temperature; Ta is ambient air temperature; ra is aerodynamic resistance.

The latent heat is estimated as

(6) $$LE=\rho Cp\frac{e_{s}-e_{a}}{\gamma (r_{a}+r_{s})}$$

where γ is the psychrometric constant (hPa/K); es is the saturation vapor pressure (hPa) at the surface temperature; and rs is the stomatal resistance (s/m), which depends on vegetation and meteorological and atmospheric conditions.

The sensible heat flux due to radiant heat balance is expressed as Hn, which can be calculated as the residue of the heat balance equation.

(7) Hn=Rn−G−LE

Finally, the sensible heat flux due to the artificial/anthropogenic (*H_as_*) effects is estimated as the difference between the total sensible heat flux and the natural sensible heat.

(8) $$H_{as}=H-Hn$$

The satellite-data-estimated LST was compared with in situ LST for 2017, whereas the estimated H_as_ was compared with in situ GWT for 2009.

**Figure 2:**
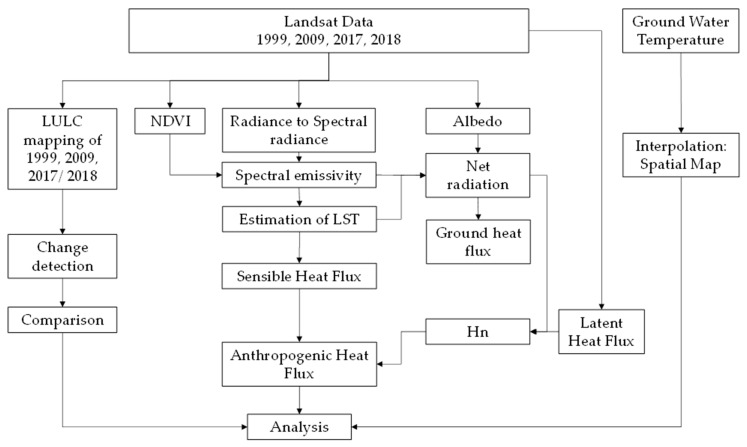
Overall data processing flowchart.

The overall methodological data processing flowchart is given in Figure 2.

## 3. Results

### 3.1. Land Use and Land Cover Mapping

**Figure 3:**
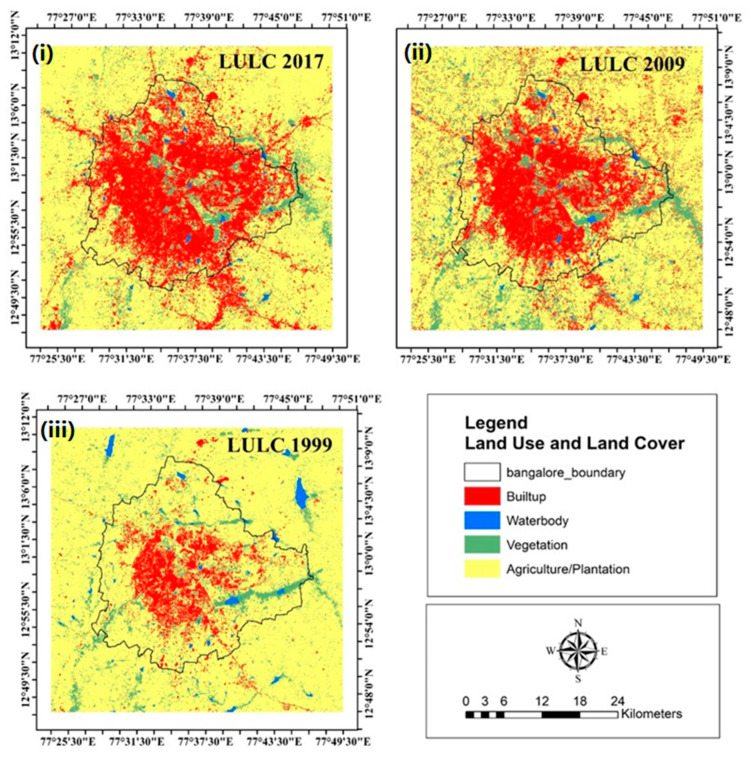
Land use/land cover (LULC) map for (**i**) 2017, (**ii**) 2009, and (**iii**) 1999.

The study analyzed LULC changes over an 18-year period. In 2009, the LULC distribution showed a concentration of built-up areas in the city center, with agricultural lands primarily located in the peripheral regions. The temporal trend revealed a clear pattern of peripheral agricultural areas being converted into built-up zones. Significant spatial changes in LULC were observed between 1999 and 2017/2018. The built-up area experienced the highest rate of change, increasing from 7.61% in 1999 to 28.36% in 2009 and slightly rising to 28.78% in 2017 (Figure 3, Table 2). This represents a nearly fourfold increase in built-up area, from 16,465.23 hectares in 1999 to 62,267.4 hectares in 2017. During the same period, vegetation cover declined by approximately 2.89% (6250.23 ha), while agricultural land showed a substantial decrease of around 17.07% (37,574.09 ha). Additionally, waterbodies decreased by approximately 1081 ha between 1999 and 2017. The expansion of built-up areas largely occurred at the expense of agricultural and plantation lands, followed by losses in vegetation and waterbodies.

### 3.2. Analysis of Land Surface Temperature (LST)

Satellite-data-derived LST estimates for 2017 were compared with in situ measurements across different LULC classes, and a close agreement was found (Table 3). However, the field-measured LST values were generally higher by more than 2 °C compared with the satellite estimates, which is likely due to differences in data acquisition times between ground measurements and satellite passes.

**Figure 4:**
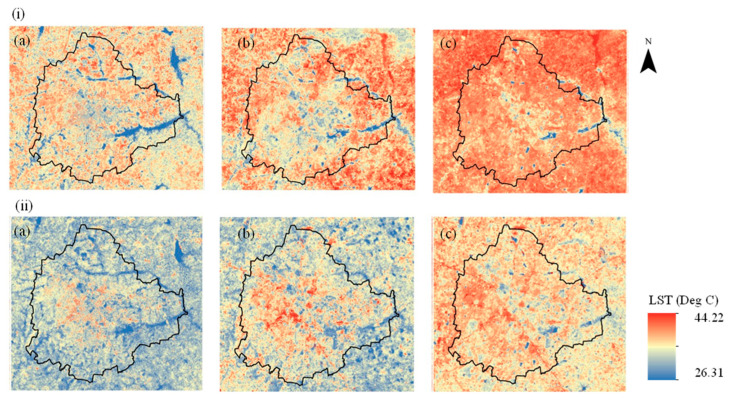
Land surface temperature (LST) estimated using Landsat data from (**a**) 1999, (**b**) 2009, and (**c**) 2017/2018 for (**i**) summer and (**ii**) winter.

The LST change map showed an increase continuously from 1999 to 2017 in both summer and winter (Figure 4, Table 4). A significant rise in LST was observed across built-up areas during both seasons. Specifically, the LST in built-up regions increased by 3.2 °C in summer and approximately 4 °C in winter between 1999 and 2017. The greatest increase in LST was recorded in agricultural and plantation areas, with a rise of about 6 °C in both summer and winter. In addition, LST increases were noted in vegetation areas (3.6 °C in summer and roughly 5 °C in winter) and waterbodies (3.65 °C in summer and approximately 3 °C in winter) over the same period. Overall, the LST rise was greater during winter than during summer for vegetation (>5 °C versus 3.6 °C) and built-up areas (~4 °C versus 3.2 °C). Conversely, there was a higher increase in LST in waterbodies in summer (3.65 °C) compared with winter (2.95 °C). The LST changes in agricultural and plantation areas remained similar in the two seasons, at around 6 °C. The changes in LST were higher during 2009–2017/2018 compared to the previous decade (1999–2009).

### 3.3. Analysis of Anthropogenic Heat Flux

**Figure 5:**
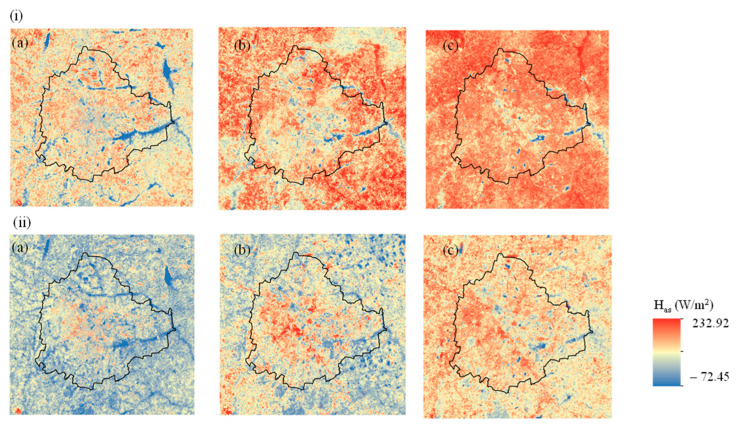
Anthropogenic heat flux (H_as_) estimated for (**a**) 1999, (**b**) 2009, and (**c**) 2017/2018 for (**i**) summer and (**ii**) winter.

The estimated anthropogenic heat flux (H*_as_*) showed an increasing trend over the years studied (Figure 5, Table 5). In 1999, H_as_ ranged from −72.45 W m^−2^ to 203.27 W m^−2^ in summer and from −9.24 W m^−2^ to 191.1 W m^−2^ in winter. By 2009, these ranges expanded to −52.59 W m^−2^ to 232.92 W m^−2^ in summer and −28.14 W m^−2^ to 215.45 W m^−2^ in winter. Further increases were observed in 2017/2018, with H_as_ ranging from 38.32 W m^−2^ to 253.14 W m^−2^ in summer and from 19.54 W m^−2^ to 220.53 W m^−2^ in winter. The highest increase in summer H_as_ occurred over built-up areas, rising from 51.33 W m^−2^ in 1999 to 119.01 W m^−2^ in 2017/2018 (Table 5). Similarly, in winter, the greatest increase in H*_as_* was also observed in built-up areas, rising from 16.38 W m^−2^ in 1999 to 93.39 W m^−2^ in 2017/2018. Agriculture and plantation areas also experienced a significant increase in H_as_, approximately 50 W m^−2^ in summer and 57 W m^−2^ in winter over the same period. In comparison, other LULC classes showed lower increases in H_as_ during summer: approximately 10 W m^−2^ in vegetation areas and 12 W m^−2^ in waterbodies. In winter, these increases were slightly higher, at about 11 W m^−2^ in vegetation areas and 19 W m^−2^ in waterbodies. Overall, the H*_as_* change from 1999 to 2017/2018 involved a comparatively greater increase in winter than in summer across all land use categories.

### 3.4. Relationship Between Anthropogenic Heat Flux (H_as_), Land Surface Temperature (LST), and Groundwater Temperature (GWT)

**Figure 6:**
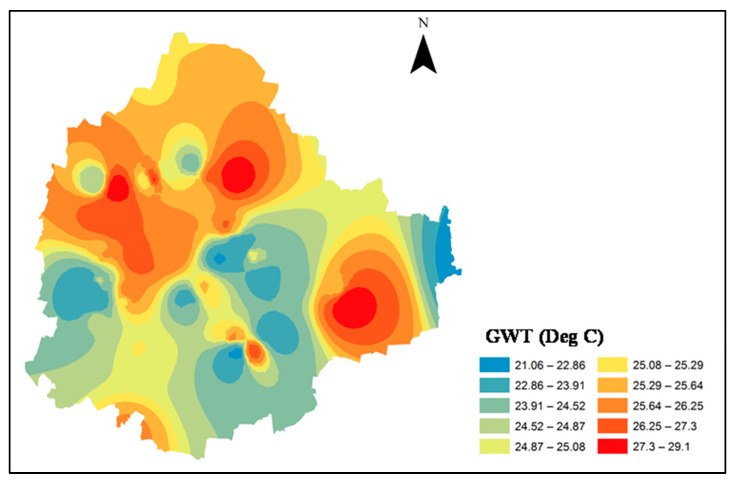
Groundwater temperature (GWT) map of Bangalore City in 2009.

**Figure 7:**
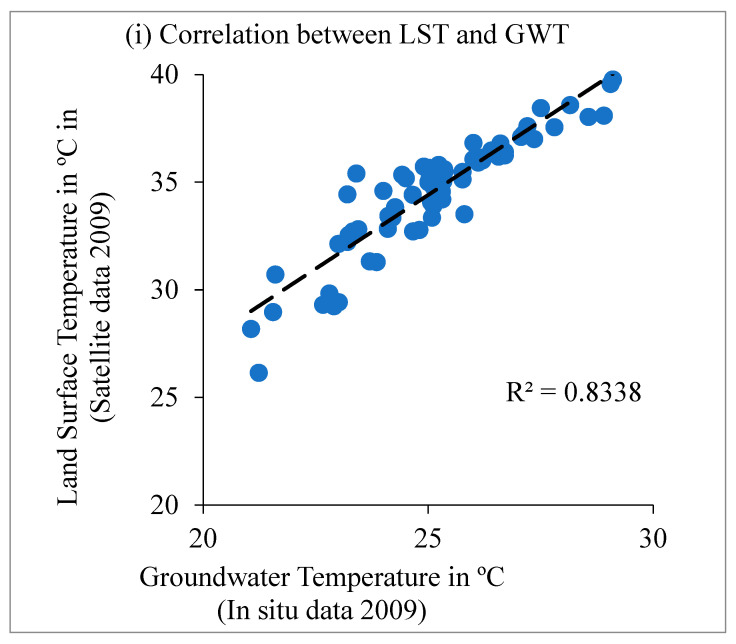

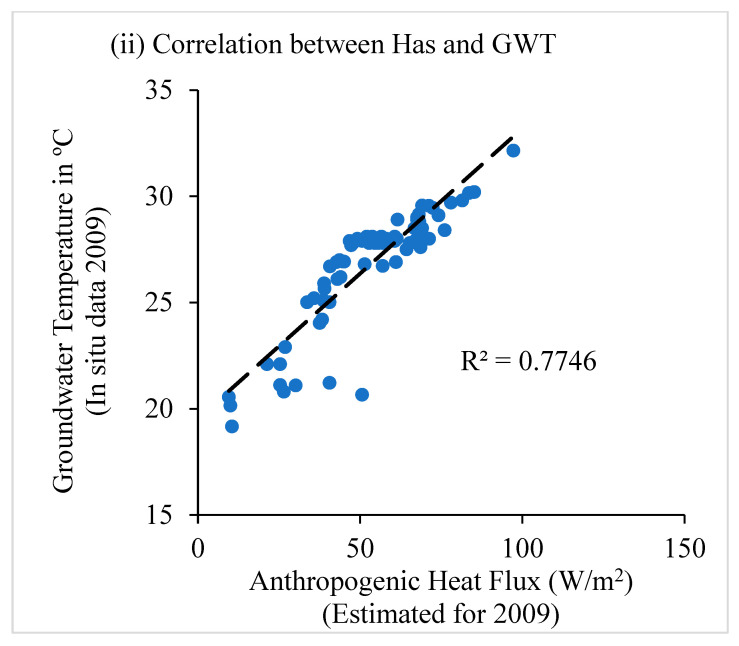
Relation between GWT (in situ data for 2009) and (**i**) LST (satellite estimates for 2009) and (**ii**) anthropogenic heat flux (H_as_ satellite estimates for 2009).

The groundwater temperature (GWT) surface map, generated using inverse distance weighting (IDW) interpolation, is presented in Figure 6. The map shows relatively higher GWT values in the northern, northwestern, and eastern regions. A linear regression analysis between H*_as_* and GWT for 2009 revealed a strong positive correlation (R^2^ > 0.83), indicating a significant influence of anthropogenic heat flux on GWT (Figure 7). The LST and in situ GWT measurements collected further showed elevated groundwater temperatures in concrete urban areas compared with regions near vegetation and waterbodies (Table 6). The borewells used for GWT data collection were generally shallow, suggesting that surface land temperature has a considerable impact on subsurface groundwater temperatures.

**Figure 8:**
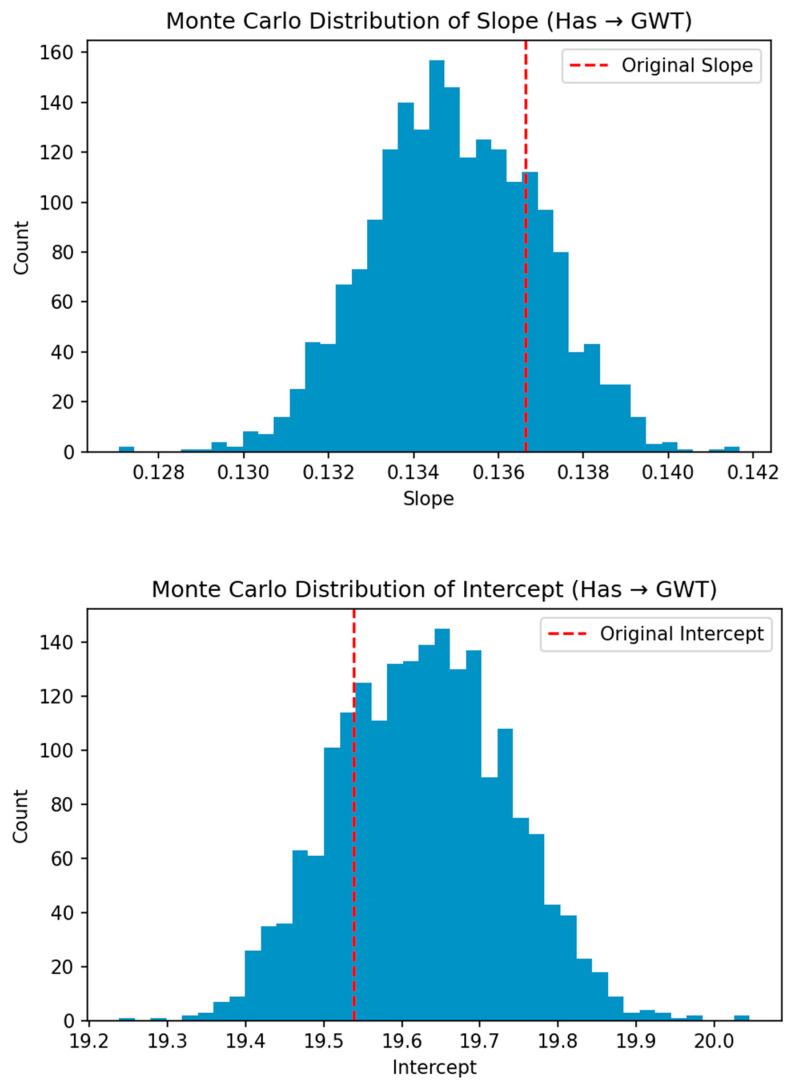
Slope and intercept values: original (red line) and Monte Carlo (MC) simulation histogram.

Monte Carlo (MC) simulation was performed assessing the sensitivity of H_as_ impact on GWT. The MC simulation was performed in Python 3.4 with ±2 units perturbation for 2000 iterations. The results are shown in Table 7 and Figure 8. As can be noted, the difference between the original slope and the intercept is low, with the MC estimated mean (MC Mean) values falling between the 5th and 95th percentile range, and the low standard error (SE) indicates a robust result. This indicates that H_as_ change would modify GWT significantly. The positive slope value of 0.14 indicates that a rise in H_as_ of 1 W m^−2^ would increase the GWT by 0.14 °C.

## 4. Discussion

The comparison of in situ LST and satellite estimates indicates that a bias of 2 °C could be attributed to the time difference between field visits (10:00 a.m. and 1:00 p.m.) and Landsat satellite pass time (10 a.m. ± 15 min). The difference indicates a rapid change in the hourly temperature during daytime in the summer month (April) in tropical regions like India. The in situ data collection partially overlapped with the peak temperature from 12 p.m. to 3 p.m. Urban sprawl was observed radiating outward in all directions from the core of Bangalore City. The built-up area growth was notably higher in the Dasarahalli region, primarily an industrial estate. The expansion in northern Bangalore can be attributed to the establishment of the New International Airport at Devanahalli. Moderate growth and increased built-up density were observed in the central part of the city. The development toward Bangalore East is linked to the formation of the International Tech Park (ITPL) in Whitefield. The western extension resulted from settlements near the Peenya Industrial Area. The southeastern part of Bangalore, especially the outer zones, such as Bommanahalli, showed the highest relative increase in built-up area, with many information technology (IT) organizations situated near Electronic City, along Hosur Road.

Bangalore’s population used to be approximately 9.6 million, with population density of about 4381 persons per square kilometer (India Census; 2011). This number has since risen to over 13 million (https://worldpopulationreview.com/world-cities/bangalore-population, accessed 3 June 2023). This population increase directly influenced LULC changes and urban sprawl in the city. The current study found that urbanization and green cover loss were greater between 1999 and 2009 compared with the more recent decade (2009–2017/2018).

Kanga et al. [29] reported a near doubling of the built-up area and an equivalent reduction in vegetation cover in Bangalore from 2001 to 2021. Their analysis showed that the temperature increased by 0.34 °C per year in urban areas compared with 0.14 °C per year in non-urban areas. Govind and Ramesh [30] noted less urban expansion in recent decades compared with the 1990s; yet, the mean LST in urban areas rose by approximately 8 °C between 1989 and 2017. Sussman et al. [31] documented a 15% increase in urban expansion from 2003 to 2018. Their analysis of MODIS daytime and night-time LST data revealed an increasing UHI trend during the dry season (December–February; night-time) and the wet season (August–October; daytime and night-time), driven by urban area growth.

Mandal and Subbaiyan [32] observed that the anthropogenic heat flux in Bangalore was highest for buildings (443.0 W m^−2^), followed by vehicular heat (87.2 W m^−2^) and metabolic heat (22.8 W m^−2^) in high-density residential areas in 2017. Lower heat flux values were estimated for low-density residential, public, semi-public, and agricultural areas. Mandal and Subbaiyan [32] reported a strong positive correlation (>0.7) between non-residential building surface fractions and anthropogenic heat flux. Ziaul and Pal [33] studied urban expansion in English Bazar town, West Bengal, India, noting a 15.27% increase in built-up area from 1990 to 2017. This growth raised the maximum anthropogenic heat flux from 54.52 W m^−2^ to 188 W m^−2^, with higher heat fluxes over impervious urban areas compared with peripheral suburban regions. In situ O_3_, NO, NO_2_, and NO_x_; concentration data for Bangalore (2015–2018) were closely correlated with vehicular emissions and planetary boundary layer dynamics, which regulate anthropogenic heat flux [34]. Benz et al. [22] reported an over 80% correlation between LST and GWT in German cities, finding that groundwater temperatures exceeded the LST due to additional subsurface heat sources. Böttcher and Zosseder [35] examined the impact of natural and anthropogenic factors on groundwater temperature in Munich city, Germany, and reported that surface sealing, aquifer thickness, and depth-to-water ratio are the major controlling factors.

The present study confirms the effectiveness of satellite remote-sensing data for analyzing the impact of LULC changes on LST and anthropogenic heat flux over 18 years in Bangalore and its surrounding areas. The key findings include the following:

- Significant growth of built-up areas, nearly fourfold, from 7.61% in 1999 to 28.77% in 2017. This growth came at the expense of agricultural/plantation lands, vegetation, and waterbodies, likely driven by population surge.
- Close agreement between satellite-derived LST values and in situ measurements. LST increased in both summer and winter, with the greatest rises observed in the agriculture/plantation and built-up classes. Increased built-up area is likely the main driver behind the rising LST and anthropogenic heat flux.
- Has increased substantially over 17 years, especially in built-up areas (greater than 77 W m^−2^ in winter and over 67 W m^−2^ in summer) and agriculture/plantation areas (approximately 57 W m^−2^ in winter and 50 W m^−2^ in summer), with lower increases in vegetation areas and waterbodies.
- Strong positive correlations (R^2^ > 0.8) between Has and GWT, as well as between LST and GWT, highlight the influence of the UHI effect on GWT.
- MC simulation results with a positive slope value (0.14) between H_as_ and GWT indicate that larger anthropogenic heat would lead to warmer groundwater.

The strong correlation (R^2^ > 0.8) between land surface temperature and anthropogenic heat flux and the high correlation (R^2^ > 0.7) between anthropogenic heat flux and groundwater temperature reflect the coupling and heat transfer between surface and subsurface features to aquifer water temperature in urban areas. The LULC change indicated rapid urbanization, which increased the impervious surfaces, such as concrete and asphalt (from 16,465 ha to 62,267 ha), replacing the permeable vegetation cover (from 24,900 ha to 18,739 ha) and waterbodies (from 2274 ha to 1026 ha). Such changes reduce water and energy transfers through infiltration and evaporative cooling. In addition, warmer nights lead to slower release of the trapped heat and lead to higher heat retention and downward heat conduction, thereby increasing the aquifer temperatures. In addition, the heat release from industrial activities, high-rise buildings, and the transport system reduces air circulation and exaggerates heat retention. Thus, the study advises using data-driven heat indicators for sustainable urban development.

The study outcome is particularly useful for urban planning and relevant policy developments, primarily focusing on

- Urban area densification: Hotspot zones with high anthropogenic heat flux should be identified to guide further construction activities, including urban area densification and vertical expansion.
- Hydrological regime and water resource development planning: Given the linkages established in this study, the groundwater extraction rules and recharge mechanisms should be revised.
- Climate-resilient urban infrastructure: The surge in urban heating is increasing the energy demand for cooling, thereby causing higher carbon and GHG emissions and further exacerbating the risk of heating [36]. Data-informed guidelines should be prepared to develop energy-efficient infrastructures.
- Biodiversity conservation and NBS: Relating urban heating with tree and water resources for prescribing NBS and to conserve and improve biodiversity [6]. Tree-based restoration can help reduce the LST, and thereby H_as_, through shading, evapotranspiration cooling, carbon sequestration, and reducing the energy demand.
- Public health and livability: The indicators of urban heating are also important in assessing the impact on public health, most importantly on vulnerable groups (children, elderly persons, pregnant women, marginalized communities, and constructions workers). A recent study has reported that several Indian cities are experiencing an increased number of very warm nights, including Bangalore, underscoring the need for using anthropogenic heat flux in urban heat mitigation planning [37].

Future research should incorporate more extensive in situ GWT data collected over multiple time periods, seasons, and depths. The approach can be applied to automated and periodic urban heating monitoring at seasonal and annual scales. In addition to the optical data, microwave data can also be employed for enhanced LULC classification, including green cover mapping [38,39,40]. Monitoring tree cover and water resources could be important components for urban development planning. Further studies may include factors like subsurface geology and aquifer characteristics. Hydrological modeling could further elucidate the complex interactions between anthropogenic activities, LST, and GWT dynamics.

## 5. Conclusions

Few studies in India have investigated the impact of urbanization on GWT. This study demonstrates the potential use of satellite remote sensing in assessing LST and anthropogenic heat flux and their influence on GWT in an urban context. The satellite-derived LST agreed closely with in situ measurements, which validates the reliability of the method. The observed increases in LST and anthropogenic heat flux, driven by urbanization and human activities, highlight an urgent need for sustainable development planning. Environmentalists, civil society organizations, and government agencies, including urban planners, should prioritize enhancing green cover and implementing tree-based restoration interventions to mitigate urban heat effects. Improving the green cover not only helps regulate the temperature but also supports biodiversity, maintains the hydrological balance, and provides vital ecosystem services. The methodology applied here can be adapted for other urban regions, climatic zones, and hydrological settings, potentially forming the basis for systematic, long-term monitoring using internet of things (IoT)-based sensor networks. Continuous monitoring of global warming trends and anthropogenic impacts on GWT will be critical for hydrologists and policymakers engaged in water resource planning, management, and informed decision making.

## Figures and Tables

**Table 1:** List of Landsat images and in situ data used in the current study.

Data	Source	1999		2009		2017/2018	
		Summer	Winter	Summer	Winter	Summer	Winter
Landsat-TM 5	USGS EarthExplorer	7 April	19 December	18 April	28 November		
Landsat-8						24 April 2017	5 January 2018
Groundwater Temperature	In situ data			14 April 2009			
Land Surface Temperature						24–25 April 2017	
Climate	IMD *	For all the Landsat dates					
Topography	SRTM-DEM ^#^	Not Applicable					

* India Meteorological Department (IMD). ^#^ Shuttle Radar Topography Mission (SRTM) Digital Elevation Model (DEM).

**Table 2:** Area (in ha and %) statistics for different LULC classes in 1999, 2009, and 2017.

LULC	1999		2009		2017	
	in ha	in %	in ha	in %	in ha	in %
Vegetation	24,989.76	11.55	18,739.53	8.66	18,739.53	8.66
Waterbody	2274.39	1.05	1193.4	0.55	1025.91	0.47
Built-up	16,465.23	7.61	61,370.54	28.37	62,267.40	28.79
Agriculture/Plantation	172,628.19	79.79	135,054.1	62.42	134,324.73	62.08

**Table 3:** Satellite and in situ LST comparison for April 2017.

LULC Classes	Date (Ground Data Collection)	Field-Collected LST (°C)	Landsat-Data-Derived LST (°C)
Vegetation	24 April 2017	31.30	29.01
Waterbody	24 April 2017	30.15	28.30
Built-up	25 April 2017	40.55	37.15
Agriculture/Plantation	25 April 2017	38.45	35.22

**Table 4:** Comparison of satellite-data-derived LST (°C) for different LULC classes.

LULC	Summer			Winter		
	April 1999	April 2009	April 2017	December 1999	November 2009	January 2018
Vegetation	26.40	26.58	30.01	23.11	25.12	28.15
Waterbody	24.15	25.37	27.80	25.22	26.17	28.17
Built-up	33.90	35.22	37.10	28.12	28.80	32.10
Agriculture/Plantation	29.10	33.12	35.17	24.22	25.46	30.15

**Table 5:** Comparison of anthropogenic heat flux (W m^−2^) for 1999, 2009, and 2017/2018.

LULC	Summer			Winter		
	April 1999	April 2009	April 2017	December 1999	November 2009	January 2018
Vegetation	15.36	17.38	25.20	−12.44	−6.29	−1.72
Waterbody	3.44	5.78	15.33	−11.51	−9.08	−30.95
Built-up	51.33	78.04	119.01	16.38	48.80	93.39
Agriculture/Plantation	24.04	15.59	73.87	−18.33	−0.76	38.63

**Table 6:** LULC comparison with GWT and LST for 2009.

Class	Groundwater Temperature (°C)	Land Surface Temperature (°C)
Built-up	27.20	37.61
Open land	29.05	39.57
Fallow land	23.20	34.44
Shrub land/parks/forest	21.20	29.37
Waterbody	21.06	28.19

**Table 7:** Monte Carlo (MC) simulation results performed for Has and GWT with 2000 iterations.

Parameter	Original Estimate	Analytical SE	MC Mean	MC SD	MC 5th	MC 95th
Slope	0.1366	0.0085	0.1350	0.0020	0.1318	0.1382
Intercept	19.5384	0.4767	19.6269	0.1089	19.4507	19.8044

## Data Availability

The data presented in this study are available on request from the corresponding author.
